# Novel genes and mutations in patients affected by recurrent pregnancy loss

**DOI:** 10.1371/journal.pone.0186149

**Published:** 2017-10-10

**Authors:** Paula Quintero-Ronderos, Eric Mercier, Michiko Fukuda, Ronald González, Carlos Fernando Suárez, Manuel Alfonso Patarroyo, Daniel Vaiman, Jean-Christophe Gris, Paul Laissue

**Affiliations:** 1 Center For Research in Genetics and Genomics-CIGGUR. GENIUROS Research Group. School of Medicine and Health Sciences, Universidad del Rosario. Bogotá, Colombia; 2 Department of Haematology, University Hospital, Nîmes. Faculty of Pharmacy and Biological Sciences and Research Team EA 2992, University of Montpellier, Montpellier, France; 3 Laboratory for Drug Discovery. National Institute of Advanced Industrial Science and Technology (AIST). Tsukuba city, Ibaraki, Japan; 4 Fundación Instituto de Inmunología de Colombia (FIDIC), Bogotá D.C., Colombia; 5 Universidad de Ciencias Aplicadas y Ambientales (UDCA), Bogotá D.C., Colombia; 6 Basic Sciences Department, School of Medicine and Health Sciences, Universidad del Rosario, Bogotá D.C., Colombia; 7 Institut Cochin, Université Paris Descartes, CNRS (UMR 8104), Paris, France; 8 Inserm, U1016, Paris, France; German Cancer Research Center (DKFZ), GERMANY

## Abstract

Recurrent pregnancy loss is a frequently occurring human infertility-related disease affecting ~1% of women. It has been estimated that the cause remains unexplained in >50% cases which strongly suggests that genetic factors may contribute towards the phenotype. Concerning its molecular aetiology numerous studies have had limited success in identifying the disease’s genetic causes. This might have been due to the fact that hundreds of genes are involved in each physiological step necessary for guaranteeing reproductive success in mammals. In such scenario, next generation sequencing provides a potentially interesting tool for research into recurrent pregnancy loss causative mutations.

The present study involved whole-exome sequencing and an innovative bioinformatics analysis, for the first time, in 49 unrelated women affected by recurrent pregnancy loss. We identified 27 coding variants (22 genes) potentially related to the phenotype (41% of patients). The affected genes, which were enriched by potentially deleterious sequence variants, belonged to distinct molecular cascades playing key roles in implantation/pregnancy biology.

Using a quantum chemical approach method we established that mutations in MMP-10 and FGA proteins led to substantial energetic modifications suggesting an impact on their functions and/or stability.

The next generation sequencing and bioinformatics approaches presented here represent an efficient way to find mutations, having potentially moderate/strong functional effects, associated with recurrent pregnancy loss aetiology. We consider that some of these variants (and genes) represent probable future biomarkers for recurrent pregnancy loss.

## Introduction

Recurrent pregnancy loss (RPL), defined as at least two/three pregnancy losses before the 20^th^ week of gestation, is a frequently occurring human infertility-related disease affecting ~0.8% to 1.4% of women in the general population [1] ^(and references therein)^. Several aetiologies and conditions have been associated with RPL, such as parental and embryo chromosomal abnormalities, prothrombotic states, uterine structural diseases, endocrinological dysfunctions, infections and immunological factors [2]. However, despite significant advances in the clinical and biochemical diagnosis of human infertility, it has been estimated that the cause remains unexplained in 35% to 60% of RPL women, thereby strongly suggesting that genetic, epigenetic and environmental factors may contribute towards the RPL phenotype [2,3].

Concerning the molecular aetiology of RPL, several studies have had limited success in identifying the disease’s genetic causes. Numerous genes (e.g. *AMN*, *THBD*, *PROCR*, *VEGF*, *TP53*, *NOS3*, *JAK2*) coding regions have been studied, in a significant number of patients, but causative mutations have rarely been described [4–7] ^(and references therein)^. Due to the natural molecular complexity of each physiological step necessary for guaranteeing reproductive success in mammals accurately targeting of the genomic regions responsible for complex traits has been challenging. Indeed, it has been shown that oocyte fecundation in mammals, as well as embryo pre-implantation, implantation and early post-implantation molecular pathways are controlled by hundreds of molecules which are finely regulated in terms of gene expression [8–11].

It has been particularly difficult to determine a priority strategy in such scenario for selecting coherent RPL candidate genes for direct sequencing. Alternative approaches, such as genome-wide association studies (GWAS) and quantitative trait loci (QTL) mapping, have been proposed for mapping loci related to the disease’s aetiology. GWAS, extensively used during the past ten years for locating chromosomal regions and SNPs associated with frequent diseases, has also been used in RPL research but it has not led to report consistent molecular markers of the disease [12–14]. Some studies using a particular congenic mouse model (the interspecific recombinant congenic strains-IRCS model), leading to QTL identification, have reported small chromosomal loci containing a limited number of candidate genes related to embryonic resorption, a phenotype analogous to human RPL [15–18]. More recently, a functional association between *FOXD1* mutations and RPL has been described [19].

In such scenario, next generation sequencing (NGS) provides a potentially interesting tool for research into RPL causative mutations. At present, NGS is a reliable technique allowing simultaneous analysis of large genomic regions in numerous patients with affordable costs.

The present study thus involved whole-exome sequencing, for the first time, in unrelated women affected by RPL. Innovative bioinformatics analysis was mainly based on studying non-synonymous sequence variants in a subset of 234 RPL candidate genes.

27 coding variants in 22 genes potentially related to the phenotype were identified in 20 out of 49 patients (41%). Using a quantum chemical approach method, we established that mutations in MMP-10 and FGA led to substantial energetic modifications suggesting an impact on protein function and/or stability. The NGS and bioinformatics approaches presented here represent an efficient way to find mutations, having potentially moderate/strong functional effects, associated with RPL aetiology. We consider that some of these variants (and genes) represent potential future biomarkers for RPL.

## Materials and methods

### Women affected by RPL

Forty-three Caucasian RPL-affected patients (Pt-1 through Pt-43) formed part of a previously-established group (the Nîmes Obstetricians and Haematologists patient cohort) of women suffering pregnancy loss [20]. Six additional Caucasian RPL patients (Pt-44 through Pt49) attended the Rosario University’s Genetics and Genomics Centre (Bogotá, Colombia). All patients displayed idiopathic RPL. They presented normal karyotypes and did not display uterine malformations, antecedents of autoimmune, coagulation and/or metabolic disorders. All cases lack familial antecedents of RPL. RPL women did not reported history of consanguinity. Patients´ average age was 34 years old. Patients had experienced 2 to 7 primary idiopathic pregnancy losses prior to the 20^th^ week of gestation. Miscarriages occurring before 10 weeks of gestation were considered embryonic (EL) while those recorded after such gestation age were classified as foetal (FL). 40 women (82%) had suffered EL and 6 FL (12%). Three patients had suffered both EL and FL (6%). **S1 Table** summarises the RPL group’s clinical characteristics. All this study's clinical and experimental steps were approved by the Institutional Ethics Committee of the Universidad del Rosario (Bogotá, Colombia) and the University of Montpellier (France) (number: ABN026-000155). The clinical investigation was performed according to the Helsinki Declaration of 1975, as revised in 1996. All the women had given their informed consent to participate.

### NGS, Sanger sequencing and bioinformatics analysis

Total DNA was extracted from patients’ blood leucocytes by conventional salting-out procedure. Experimental details of library preparation, sequencing and bioinformatics analysis have been included as **S1 Methods**. **S2 Table** includes the subset of 234 RPL (RPL-234) candidate genes used for bioinformatics analysis. The **S1 Fig** describes the methodological pipeline for creating the 234-RPL subset.

### Structure preparation/modelling and Fragment Molecular Orbital (FMO) calculations

Experimental details of protein structural analysis have been included as **S1 Methods.**

### Statistical analyses

Comparisons between the RPL-234 subset of genes and the rest of the genome were performed using a contingency χ^2^ test.

## Results

### Sequencing and bioinformatics analysis

All samples’ alignment ranged from 85%–98%. Almost 98% of the target at 1X depth was covered for all samples and more than 85% of the target was covered at 30X depth. Exome data was uploaded to the NIH Short Read Archive repository (https://www.ncbi.nlm.nih.gov/sra) (BioProject accession number: PRJNA325830; Short Read Archive- SRA accession number: SRP114508).

**Fig 1:**
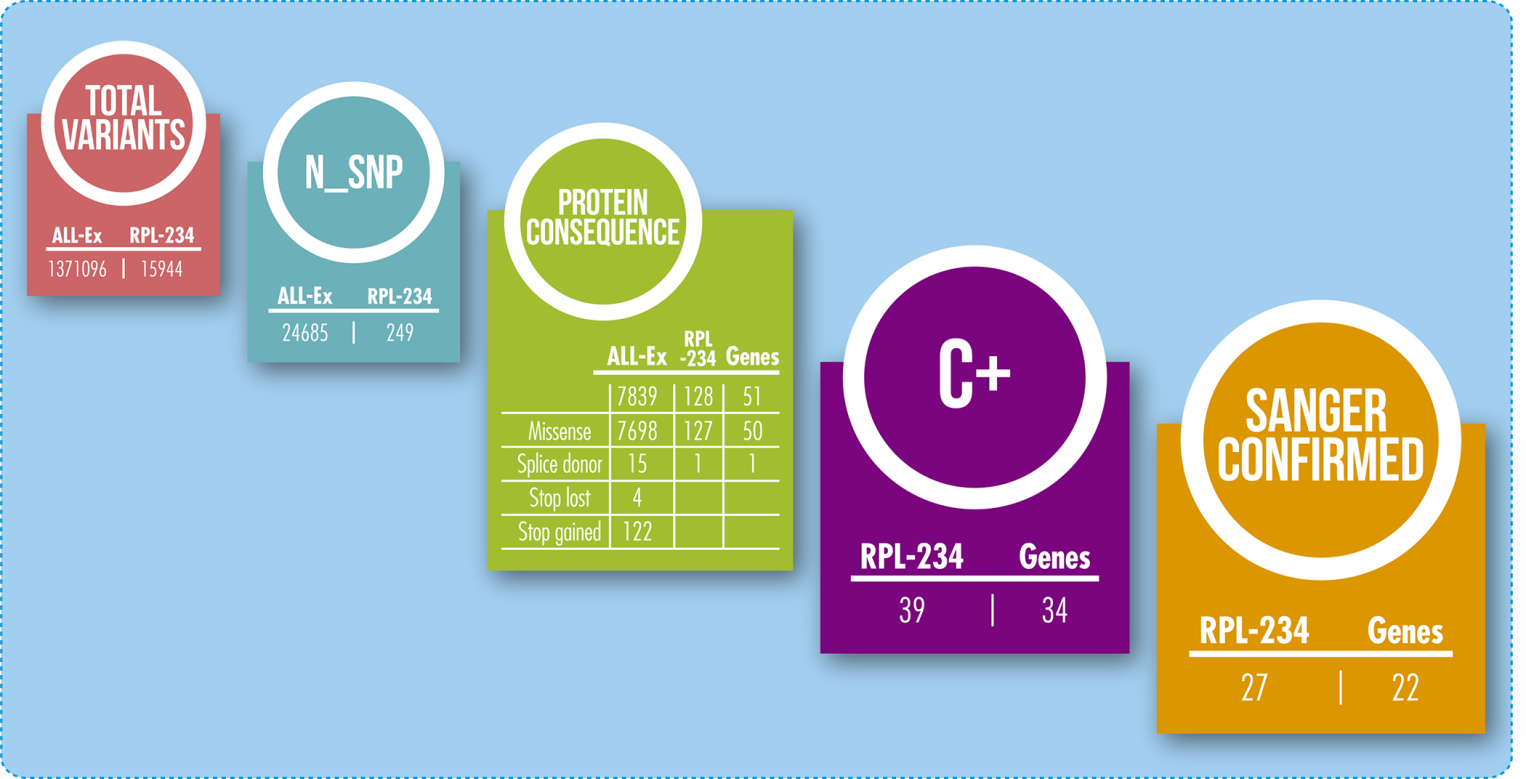
Bioinformatics filtering of exome sequencing data. **All-ex:** Complete exome sequencing data excluding RPL-234 results. **RPL-234:** group of RPL candidate genes. **C+:** sequence variants involving residues strictly conserved during evolution.

RPL-234 data analysis revealed 15944 sequence variants (**Fig 1**). 249 of these were novel (24685 when all the genes/exons were considered throughout the genome). One splice-site variant was found which was not confirmed by direct sequencing (false positive). Among novel RPL-234, 128 variants (51%) induced a protein change while 7839 (31.7%) affected the rest of the genome (All-Ex). The statistical comparative analysis of these proportions revealed that the RPL-234 list is strongly enriched by this kind of variants (128 variants observed with 99 expected, p = 0.0005).

One hundred and twenty-seven heterozygous missense mutations were detected; 39 of them had led to changes in residues which had been conserved during evolution (C+). Twenty-seven **C+** variants, affecting 20 patients, were positively verified via Sanger sequencing and considered strong candidates related to RPL molecular aetiology (**Fig 1, Table 1**). Sixteen (80%) out 20 RPL patients carrying aetiological candidate mutations were affected by EL while 10% (2/20) displayed FL. The rest of patients (10%) was affected by EL and FL.

Score values of potential deleterious effects calculated by 4 computational prediction programs are summarized in **Table 2**.

**Table 1:** Sequence variants identified in RPL patients.

Patient ID	Gene	Mutation		Biological process
		CDS position	Protein position	
Pt-37	***TRO***	c.2909G>A	p.Gly970Asp	Cell adhesion-trophoblast endometrium interaction
Pt-39	***CDH11***	c.1355C>G	p.Ala452Gly	
Pt-20	***CDH1***	c.164T>G	p.Val55Gly	
Pt-46	***THBD***	c.457T>G	p.Trp153Gly	Coagulation
Pt-22	***F5***	c.4619A>C	p.Glu1540Ala (p.Glu1512Ala)	
Pt-22	***F5***	c.5932A>C	p.Thr1978Pro (p.Thr1950Pro)	
Pt-16	***FGA***	c.2054T>G	p.Phe685Cys	
Pt-45	***MMP10***	c.595G>A	p. Asp199Asn	Extracellular matrix remodeling
Pt-26	***MMP9***	c.17C>T	p.Pro6Leu	
Pt-28	***COL6A3***	c.6859C>T	p.Arg2287Trp	
Pt-29	***ADAMTS1***	c.2068G>C	p.Val690Leu	
Pt-45	***TNC***	c.4063G>A	p.Val1355Met	
Pt-35	***FLT1***	c.953C>T	p.Ser318Leu	Angiogenesis
Pt-31	***FLT1***	c.2435G>A	p. Arg812Gln	
Pt-5	***EPAS1***	c.1510C>G	p.Leu504Val	
Pt-11 / Pt-17	***EPAS1***	c.1463A>G	p.Tyr488Cys	
Pt-4	***LIFR***	c.2338C>T	p.Arg780Cys	Cell proliferation, differentiation, migration, apoptosis
Pt-36	***FGFR2***	c.1088C>T	p.Ala363Val	
Pt-19	***BMP7***	c.448C>T	p.Arg150Cys	
Pt-19	***AMN***	c.207G>A	p.Met69Ile	Metabolism
Pt-5	***IDO2***	c.539T>G	p.Phe180Cys	Immunological function modulation
Pt-48	***IDO2***	c.749T>C	p.Phe250Ser	
Pt-26	***CR1***	c.4501A>G	p.Thr1501Ala	
Pt-28	***TLR3***	c.2384C>T	p.Ala795Val	
Pt-20	***TRAF3IP1***	c.415C>T	p.Arg139Trp	
Pt-14	***NCOA1***	c.2011T>G	p.Ser671Ala	Steroidal nuclear receptors activation

**Table 2:** Pathogenicity predictions.

Gene	Protein position	SIFT	Polyphen	Mutation taster			Mutpred	Total
				PhyloP	PhastCons	Prediction		
**TRO**	p.Gly970Asp	Tolerated (0.07)	Probably damaging (0.999)	1.224	0.019	Polymorphism	0.379	1/4
**CDH11**	p.Ala452Gly	Tolerated (0.38)	Benign (0.197)	3.39	0.999	Disease causing	0.322	1/4
**CDH1**	p.Val55Gly	Deleterious (0)	Probably damaging (1)	3.938	1	Disease causing	0.821	4/4
**THBD**	p.Trp153Gly	Deleterious (0)	Probably damaging (0.976)	3.847	0.987	Disease causing	0.716	4/4
**F5**	p.Thr1978Pro	Deleterious (0)	Benign (0.388)	3.052	1	Disease causing	0.889	3/4
	p.Glu1540Ala	Deleterious (0.02)	Benign (0.185)	2.084	0.221	Polymorphism	0.455	1/4
**FGA**	p.Phe685Cys	Deleterious (0)	Probably damaging (1)	5.158	1	Disease causing	0.960	4/4
**MMP10**	p. Asp199Asn	Deleterious (0)	Probably damaging (0.992)	5.238	1	Disease causing	0.711	4/4
**MMP9**	p.Pro6Leu	Tolerated (0.4)	Possibly damaging (0.824)	0.878	0.366	Polymorphism	0.461	1/4
**COL6A3**	p.Arg2287Trp	Deleterious (0.02)	Probably damaging (0.985)	1.479	0.003	Polymorphism	0.517	3/4
**ADAMTS1**	p.Val690Leu	Tolerated (0.1)	Benign (0.113)	4.077	1	Disease causing	0.547	2/4
**TNC**	p.Val1355Met	Tolerated (0.06)	Benign (0.316)	1.239	0.843	Disease causing	0.674	2/4
**FLT1**	p.Ser318Leu	Tolerated (0.16)	Possibly damaging (0.794)	2.463	0.019	Polymorphism	0.472	1/4
	p.Arg812Gln	Tolerated (0.16)	Benign (0.407)	6.18	1	Disease causing	0.334	1/4
**EPAS1**	p.Tyr488Cys	Tolerated (0.08)	Possibly damaging (0.687)	3.174	1	Disease causing	0.254	2/4
	p.Leu504Val	Deleterious (0)	Probably damaging (0.979)	5.684	1	Disease causing	0.456	3/4
**LIFR**	p.Arg780Cys	Tolerated (0.07)	Possibly damaging (0.542)	1.265	0.899	Polymorphism	0.577	2/4
**FGFR2**	p.Ala363Val	Tolerated (0.34)	Possibly damaging (0.492)	4.295	1	Disease causing	0.258	2/4
**BMP7**	p.Arg150Cys	Deleterious (0)	Probably damaging (0.952)	2.863	0.998	Disease causing	0.658	4/4
**AMN**	p.Met69Ile	Tolerated (0.05)	Benign (0.016)	2.147	0.98	Disease causing	0.614	2/4
**IDO2**	p.Phe180Cys	Deleterious (0)	Probably damaging (1)	3.591	0.978	Disease causing	0.936	4/4
	p.Phe250Ser	Deleterious (0)	Probably damaging (0.994)	2.707	1	Disease causing	0.795	4/4
**CR1**	p.Thr1501Ala	Tolerated (0.3)	Benign (0.076)	-0.206	0	Polymorphism	0.540	1/4
**TLR3**	p.Ala795Val	Tolerated (0.14)	Probably damaging (0.961)	4.188	1	Disease causing	0.716	3/4
**TRAF3IP1**	p.Arg139Trp	Tolerated (0.07)	Probably damaging (0.928)	0.261	0.006	Polymorphism	0.603	2/4
**NCOA1**	p.Ser671Ala	Tolerated (0.64)	Benign (0.041)	0.447	0.997	Polymorphism	0.270	0/4

It is worth mentioning that mutations identified in F5 had originally been reported by our bioinformatics analysis as F5 p.Glu1540Ala and F5 p.Thr1978Pro (compared to the F5 NP_000121.2 sequence). However, most studies published to date have reported F5 amino acid positions following the original description by Jenny et al., in which signal peptide residues (Met^1^ through Ala^28^) are not considered for consecutive numbering. The first amino acid (+1) in their sequence is Ala^29^ [21]. We have thus used the numbering reported by Jenny et al., in the present study to enable comparing our results with other studies. F5 p.Glu1540Ala and F5 p.Thr1978Pro mutations (NP_000121.2 sequence) in the present work have thus been described as F5 p.Glu1512Ala and F5 p.Thr1950Pro, respectively.

### Fragment Molecular Orbital (FMO) analysis

**Fig 2:**
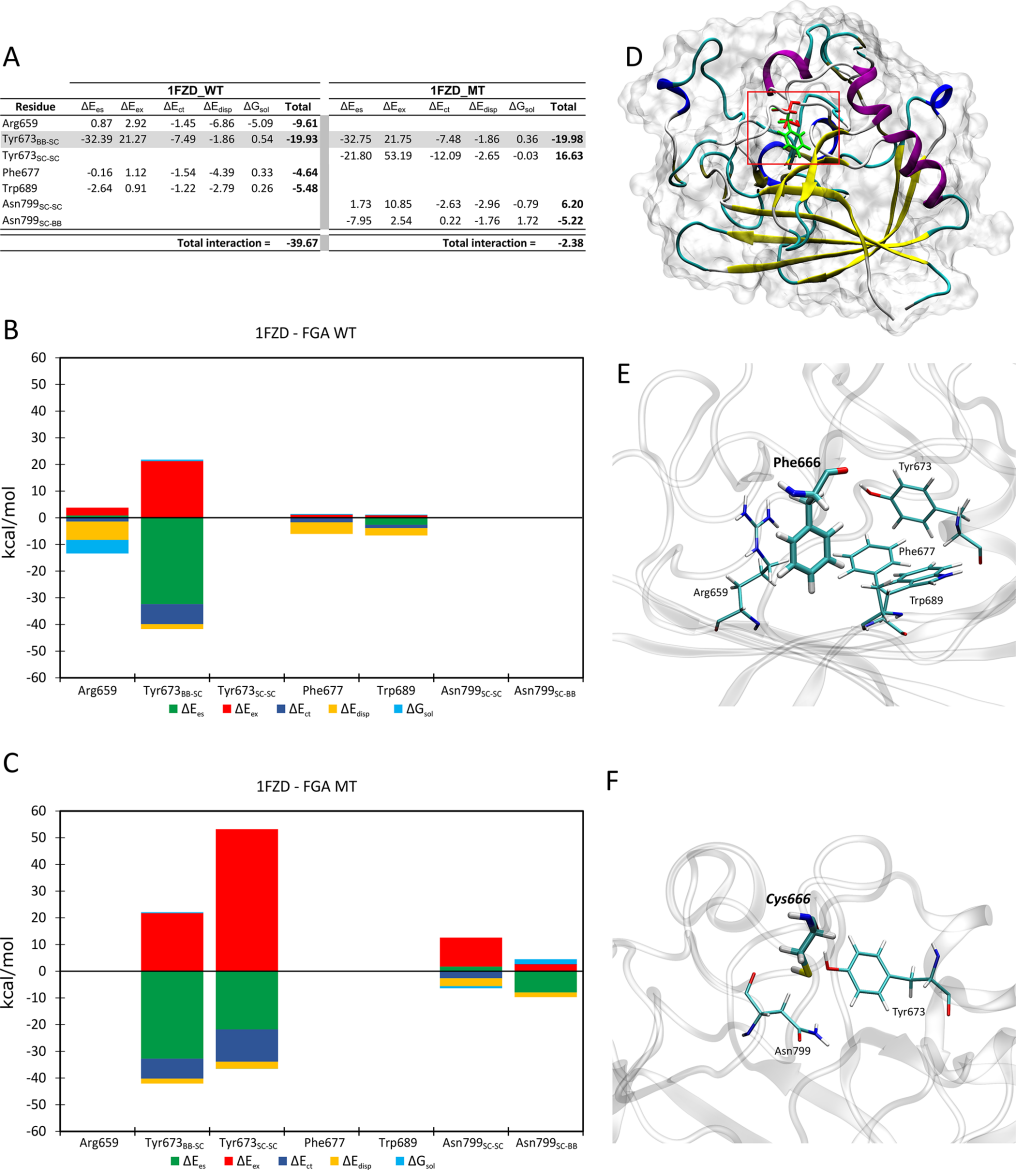
FMO results for FGA WT *vs*. MT. **(A)** Pair interaction decomposition analysis (PIEDA) contributions of amino acids interacting with position 199. Conserved interactions between WT and MT are shadowed in gray (energies are expressed in kcal/mol). **(B) WT and (C) MT.** Bar plots describe the PIEDA of energy interaction terms: electrostatics (green), exchange-repulsion (red), charge-transfer (blue), dispersion (yellow), and solvation (cyan). Positive values are considered destabilising and negative stabilising. (**D)** Overall view of the analysed system. In the red box a detail of the mutation zone. (**E)** Detail of the amino acids interacting with Phe666 in FGA WT. **(F)** Detail of the amino acids interacting with Cys666 in FGA WT. The phenylalanine mutated residue located at position 685 corresponds, into the crystallographic structure, to the amino acid located at position 666. Therefore, for FMO approach we have used Phe666 instead Phe685.

**Fig 3:**
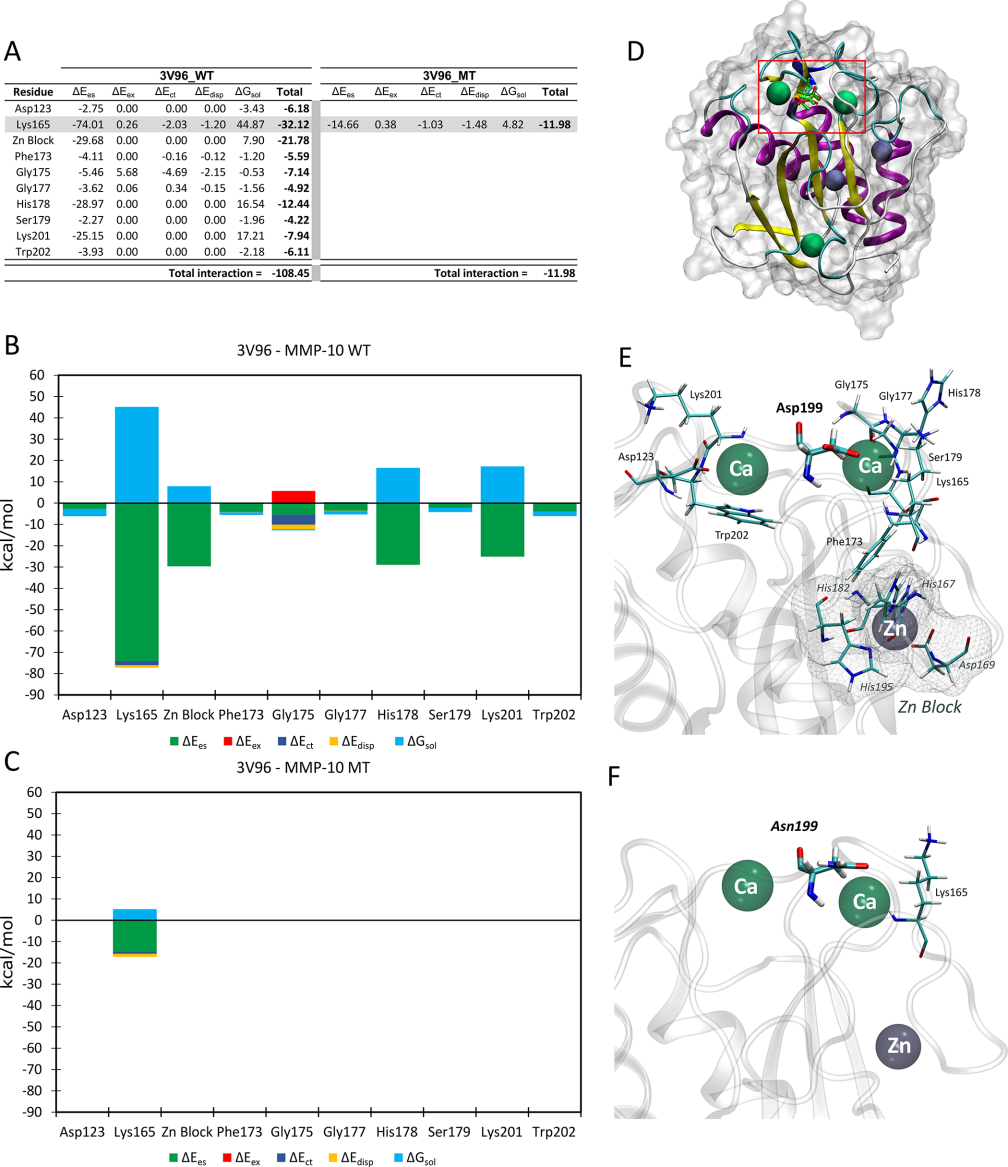
FMO results for MMP-10 WT *vs*. MT. **(A)** PIEDA contributions of amino acids interacting with position 199, conserved interactions between WT and MT are shadowed in gray (energies are expressed in kcal/mol). **(B) WT and (C) MT.** Bar plots describe the PIEDA of energy interaction terms: electrostatics (green), exchange-repulsion (red), charge-transfer (blue), dispersion (yellow), and solvation (cyan). Positive values are considered destabilising and negative stabilizing. (**D)** Overall view of the analysed system. In the red box, a detail of the mutation zone. (**E)** Detail of the amino acids and ions interacting with the Asp199 in MMP-10 WT. **(F)** Detail of the amino acids and ions interacting with the Asn199 in MMP-10.

The FMO method was used for studying FGA (fibrinogen alpha chain) (**Fig 2**) and MMP-10 (matrix metallopeptidase 10) (**Fig 3**) WT and MT structures regarding the effect of amino acid substitutions. We have selected these proteins for structural analysis because regions carrying putative deleterious mutations were previously crystallized. We have found significant changes in protein stability secondary to FGA- p.Phe685Cys and MMP10-p.Asp199Asn mutations, which strongly suggest a deleterious effect leading to RPL.

Figs 2A and 3A show the calculated values for FGA and MMP-10 models, respectively. Detailed information on results from FMO analysis has been included as **S1 Results.**

## Discussion

The present work involved whole-exome sequencing in 49 non-related patients affected by RPL. To increase the chances to find potential RPL causative mutations our attention was focused on a subset of 234 candidate genes (RPL-234). The list included genes participating in molecular cascades involved in the distinct stages of pregnancy (**S2 Table**). Since our patients were unrelated, stringent filters (e.g. novel, non-synonymous, C+) were used in putative aetiological mutation research, enabling the selection of rare variants having (hypothetically) moderate/strong functional effects. This approach led us to identify 39 heterozygous sequence variants, 27 of which (affecting 22 genes) were definitely validated by Sanger sequencing (**Table 1, Fig 1**). These variants affected 41% of patients, thereby highlighting the fact that NGS is an efficient tool for screening the genetic causes of complex reproductive disorders. Our statistical analysis revealed that the RPL-234 subgroup of genes was enriched (p = 0.0005) by variants underlying potential functional effects (mutations leading to protein sequence alteration). These findings corroborated that the analysis of a well selected group of genes might be a powerful approach for dissecting complex diseases´ molecular aetiology (see below). In a recent work Fonseca et al. reported using of custom target sequencing microarrays, in unrelated patients affected by premature ovarian failure, for identifying the genetic origin of this disorder [22]. In that study, which was performed on 70 candidate genes, 25% of patients presented potential deleterious mutations.

The present study determined that genes carrying C+ mutations were involved in the following biological processes: cell adhesion (e.g. trophoblast/endometrium interaction), coagulation, angiogenesis, immunological function response/modulation, metabolism (e.g. B12 vitamin), extracellular matrix remodelling, steroidal nuclear receptor activation and specific cell function regulation (proliferation, differentiation, migration and apoptosis) (**Table 1**).

Concerning cell adhesion molecules, TRO (trophinin) holds particular interest since it has been demonstrated to perform key functions during embryo implantation [23,24] ^(and references therein)^. Pt-37 displayed the TRO p.Gly970Asp mutation in our present study which affected an evolutionary conserved amino acid located in the decapeptide tandem repeat domain. Due to natural physicochemical differences between Gly and Asp residues, the p.Gly970Asp mutation might modify the protein’s local or global properties (e.g. folding and binding) which might perturb tastin/trophinin/bystin complex formation leading to embryo implantation disturbances and RPL. NGS and filtering analysis led to identifying four mutations in three genes involved in coagulation molecular pathways: coagulation factor V-*F5* c.4619A>C (p.Glu1512Ala), *F5* c.5932A>C (p.Thr1950Pro), thrombomodulin-*THBD* c.457T>G (p.Trp153Gly) and fibrinogen alpha chain-*FGA* c.2054T>G (p.Phe685Cys).

Although controversial results have been published to date regarding F5 and RPL origin, it can be considered a coherent candidate due to its inherent function during coagulation [25,26] ^(and references therein)^. The F5-p.Glu1512Ala mutation (Pt-22) is located in the B protein domain which encompasses amino acids Ser^710^–Arg^1545^ [27] (considerations regarding F5 residue numbering have been included in the results section). It has been shown that the residue Arg^1545^ has a large impact on F5-F10 interaction which is necessary for generating fully-activated F5, probably during late coagulation stages (e.g. when thrombin levels are still low) [27,28]. Furthermore, the B domain contains an acidic region (AR) between the Thr^1493^ and Asp^1537^ residues interacting with a basic region to keep F5 as an inactive form (procofactor) [29]. It has been shown that the Asp^1508^-Tyr^1515^ region is necessary for procofactor cleavage by thrombin and light chain formation [30]. It can thus be assumed that the C-terminal region of the B domain (e.g. AR and Arg^1545^) is crucial for protein activation/inactivation and guaranteeing proper equilibrium between F5 procoagulant and anticoagulant properties in normal conditions. The second mutation, also carried by Pt-22, which we have identified in F5 (p.Thr1950Pro), is located in the protein’s C1 domain near to functionally-relevant amino acids (Tyr^1956^, Leu^1957^) necessary for prothrombinase complex (PC) assembly on phosphatidylserine membranes and thrombin production regulation [31]. The p.Thr1950Pro mutation could interfere with normal PC function, leading to changes in thrombin bioavailability and coagulation imbalance. As already described for further F5 missense mutations, p.Glu1512Ala and p.Thr1950Pro might also be related to abnormal cytoplasmic retention, thereby contributing towards the phenotype [27].

THBD is a cell plasma membrane glycoprotein which is mainly expressed in blood vessels and placental syncytiotrophoblast participating in distinct biological processes, such as coagulation, complement activation, fibrinolysis, inflammation and cell proliferation [32–34]. THBD has been described as an anticoagulant factor by enhancing protein C activation and decreasing thrombin specificity [35]. Mice lacking *Thbd* die secondary to developmental failure of placenta associated with abnormal activation of blood coagulation cascade at the foetal-maternal interface [36,37]. The THBD-p.Trp153Gly mutation that we have identified in Pt-46 is located at the end of the C-type lectin-like module (the protein’s N-terminal region) which has mainly been linked with anti-inflammatory activity and cell adhesion [33] ^(and references therein)^. The THBD-p.Trp153Gly mutant protein might therefore contribute to placental endothelial dysfunction and/or coagulation imbalance.

FGA encodes the fibrinogen alpha subunit, a glycoprotein having several functions during coagulation physiology, such as fibrin clot formation, non-substrate thrombin binding, platelet aggregation and fibrinolysis. *FGA* mutations, especially drastic biallelic variants, have been associated with afibrinogenemia, a rare bleeding disorder [38,39]. *FGA* heterozygous missense mutations have been identified in patients affected by dysfibrinogenemia, a blood disorder having an unpredictable clinical phenotype due to abnormal fibrinogen quality [39,40]. Similarly to the variants described above affecting coagulation physiology, FGA-p.Phe685Cys mutation in Pt-16 may affect protein folding, thus leading to functional disturbances. In this case, substituting a phenylalanine for a cysteine in position 685 implies a particularly drastic change in terms of physicochemical properties.

Regarding the FMO analysis, it is important to note that we used FGA and MMP-10 proteins for structural analysis because previous studies of the affected regions (based on cristallization assays) revealed reliable/exploitable data. The FGA modelled mutation (Phe to Cys in position 666) involved changes in stabilising interactions (**Fig 2**). The mutation resulted in a major change in total interaction energy from -39.67 (WT) to -2.38 (MT) kcal/mol (almost 20-fold). The loss of three non-classical H-bonds (CH-π interactions) contributed to FGA MT protein destabilisation.

Similarly, further analyses revealed changes of one order of magnitude concerning stabilising interactions between wild-type (MMP-10 WT) and mutant (MMP-10 MT) forms of matrix metallopeptidase 10 (MMP-10) in total interaction energies (-108.45 kcal/mol WT vs. -11.98 kcal/mol MT). MMP-10 Asn199 MT lost all but one stabilising interaction, including strong interactions, such as a salt bridge (Lys165) and the block containing structural Zn^2+^ (**Fig 3**). These findings strongly suggested a significant change in protein stability. Interestingly, Pt-45 displayed 3 early losses which is coherent with the key role of MMP-10 during the invasive process of implantation [41,42]. In conclusion, our computational chemistry calculations of MMP-10 and FGA mutations thus argued strongly in favour of pathogenic effects.

Regarding variants belonging to additional biological processes IDO2-p.Phe180Cys, IDO2-p.Phe250Ser, LIFR-p.Arg780Cys, TLR3-p.Ala795Val, EPAS1-p.Tyr488Cys and EPAS1-p.Leu504Val are particularly interesting because they affected factors (indoleamine 2,3-dioxygenase–IDO2, leukemia inhibitory factor receptor alpha-LIFR, toll like receptor 3-TLR3, endothelial PAS domain protein 1-EPAS1) playing key roles during pregnancy and/or due to the mutations’ molecular nature. Although screening large cohorts of RPL individuals is necessary, the EPAS-p.Tyr488Cys mutation might represent a mutational hotspot as it was present in two unrelated patients.

In the present study, we have used 4 bioinformatic tools to predict pathogenicity caused by C+ mutations. We have chosen these programs as they have demonstrated high levels of positive prediction values [43,44]. Seven out 26 (27%) mutations displayed scores compatible with deleterious effects in all tested programs: CDH1-p.Val55Gly, THBD-p.Trp153Gly, FGA-p.Phe685Cys, BMP7-p.Arg150Cys, IDO2-p.Phe180Cys, IDO2-p.Phe250Ser and MMP-10-p.Asp199Asn (**Table 2**).

Interestingly, 35% of patients (Pt-5, Pt-19, Pt-20, Pt-22, Pt-26, Pt-28 and Pt-45) had two direct sequencing confirmed C+ sequence variants. The genes affected by these mutations in Pt-5, Pt-19 and Pt-20 are known to have functions in distinct biological processes. However, both mutations carried by Pt-22 or Pt-45 involved genes participating in common biological processes: Pt-22 had two variants in F5 (coagulation) while Pt-45 presented variants in MMP-10 and TNC (extracellular matrix remodelling). Furthermore, Pt-26 and Pt-28 were carriers of mutations present in genes (Pt-26: MMP-9 and CR1; Pt-28: COL6A3 and TLR3) affecting the same biological processes (extracellular matrix remodelling and immunological function modulation). These findings thus suggest that distinct variants’ additive/epistatic effects contribute towards RPL aetiology. Indeed, physiological (and pathological) reproductive phenotypes can be considered quantitative traits resulting from the subtle interaction of hundreds of genes. This scenario, as well as the fact that infertility genetic causative variants are under strong negative selection, evokes that reproductive biological processes are modulated by functionally-redundant molecular cascades.

Concerning segregation analysis of potentially functional variants we did propose to most of our RPL patients the idea of contacting their parents regarding their participation in our study but, unfortunately, they decided not to involve their families.

Taken together, our findings add valuable information regarding the maternal molecular origin of RPL and must be the starting point for further functional *in vitro* and *in vivo* studies. To note, RPL has to be considered as the result of a complex combination of maternal/paternal factors, thereby molecular exploration of potential paternal dysfunctions is needed [45]. The bioinformatics analysis presented here represents an efficient approach of identifying mutations (having potentially moderate/strong functional effects) associated with RPL aetiology. We hope that some of these variants (and genes) will be used, after their definitive functional validation, as RPL biomarkers.

## Supporting information

S1 Fig Methodological pipeline for creating the 234-RPL subset. (TIF)

S1 Table Clinical features of RPL patients. (DOC)

S2 Table Gene subset RSA-234. (DOC)

S1 Methods Library preparation and sequencing. (DOCX)

S1 Results Fragment Molecular Orbital (FMO) calculations. (DOCX)
