# Supplementary material for: Novel genes and mutations in patients affected by recurrent pregnancy loss
Source: PLoS One. 2017 Oct 10;12(10):e0186149. doi: 10.1371/journal.pone.0186149 (PMC5634651; doi:10.1371/journal.pone.0186149)
Supplement: S1 Table — (DOC) [file pone.0186149.s002.doc]

**Novel genes and mutations in patients affected by recurrent pregnancy loss**

Paula Quintero-Ronderos, Eric Mercier, Michiko Fukuda, Ronald González, Carlos Fernando Suárez, Manuel Alfonso Patarroyo, Daniel Vaiman , Jean-Christophe Gris and Paul Laissue

**Table S1-Clinical features of RPL patients**

| **Table S1-Clinical features of RPL patients** | | | | |
| --- | --- | --- | --- | --- |
| **Patient code** | **Total number of miscarriage** | **EL** | **FL** | **Genes and mutations** |
| Pt-1 | 4 | 4 |  |  |
| Pt-2 | 3 | 3 |  |  |
| Pt-3 | 5 | 5 |  |  |
| Pt-4 | 3 | 3 |  | LIFR - p.Arg780Cys |
| Pt-5 | 3 | 3 |  | EPAS1 - p.Leu504Val |
|  |  |  |  | IDO2 - p.Phe180Cys |
| Pt-6 | 3 | 3 |  |  |
| Pt-7 | 3 | 3 |  |  |
| Pt-8 | 3 | 3 |  |  |
| Pt-9 | 3 | 3 |  |  |
| Pt-10 | 3 | 3 |  |  |
| Pt-11 | 3 | 3 |  | EPAS1 - p.Tyr488Cys |
| Pt-12 | 3 | 3 |  |  |
| Pt-13 | 3 | 3 |  |  |
| Pt-14 | 3 | 3 |  | NCOA1 - p.Ser671Ala |
| Pt-15 | 3 | 3 |  |  |
| Pt-16 | 3 | 3 |  | FGA - p.Phe685Cys |
| Pt-17 | 3 | 3 |  | EPAS1 - p.Tyr488Cys |
| Pt-18 | 3 | 2 | 1 |  |
| Pt-19 | 3 | 3 |  | BMP7 - p.Arg150Cys |
|  |  |  |  | AMN - p.Met69Ile |
| Pt-20 | 3 | 3 |  | CDH1 - p.Val55Gly |
|  |  |  |  | TRAF3IP1 - p.Arg139Trp |
| Pt-21 | 3 | 3 |  |  |
| Pt-22 | 3 | 3 |  | F5 - p.Thr1978Pro |
|  |  |  |  | F5 - p.Glu1540Ala |
| Pt-23 | 3 | 3 |  |  |
| Pt-24 | 3 | 3 |  |  |
| Pt-25 | 3 | 3 |  |  |
| Pt-26 | 3 | 3 |  | MMP9 - p.Pro6Leu |
|  |  |  |  | CR1 - p.Thr1501Ala |
| Pt-27 | 3 | 3 |  |  |
| Pt-28 | 3 | 3 |  | COL6A3 - p.Arg2287Trp |
|  |  |  |  | TLR3 - p.Ala795Val |
| Pt-29 | 3 | 3 |  | ADAMTS1 - p.Val690Leu |
| Pt-30 | 4 | 4 |  |  |
| Pt-31 | 4 | 4 |  | FLT1 - p. Arg812Gln |
| Pt-32 | 2 |  | 2 |  |
| Pt-33 | 3 |  | 3 |  |
| Pt-34 | 2 |  | 2 |  |
| Pt-35 | 2 |  | 2 | FLT1 - p.Ser318Leu |
| Pt-36 | 4 | 4 |  | FGFR2 - p.Ala363Val |
| Pt-37 | 2 |  | 2 | TRO - p.Gly970Asp |
| Pt-38 | 3 | 3 |  |  |
| Pt-39 | 4 | 4 |  | CDH11 - p.Ala452Gly |
| Pt-40 | 3 | 3 |  |  |
| Pt-41 | 3 | 3 |  |  |
| Pt-42 | 3 | 3 |  |  |
| Pt-43 | 4 | 4 |  |  |
| Pt-44 | 18 |  | 18 |  |
| Pt-45 | 3 | 3 |  | MMP10 - p. Asp199Asn |
|  |  |  |  | TNC - p.Val1355Met |
| Pt-46 | 3 | 2 | 1 | THBD - p.Trp153Gly |
| Pt-47 | 4 | 4 |  |  |
| Pt-48 | 4 | 3 | 1 | IDO2 - p.Phe250Ser |
| Pt-49 | 4 | 4 |  |  |
