# Supplementary material for: Novel genes and mutations in patients affected by recurrent pregnancy loss
Source: PLoS One. 2017 Oct 10;12(10):e0186149. doi: 10.1371/journal.pone.0186149 (PMC5634651; doi:10.1371/journal.pone.0186149)
Supplement: S2 Table — (DOC) [file pone.0186149.s003.doc]

**Novel genes and mutations in patients affected by recurrent pregnancy loss**

Paula Quintero-Ronderos, Eric Mercier, Michiko Fukuda, Ronald González, Carlos Fernando Suárez, Manuel Alfonso Patarroyo, Daniel Vaiman , Jean-Christophe Gris and Paul Laissue

**Table S2. Gene subset RPL-234**

| **Table S2. Gene subset RPL-234** | | |
| --- | --- | --- |
| **Gene** | **Gene name** | **Gene ID** |
| *ACE* | Angiotensin I converting enzyme | NM_000789.3 |
| *ACHE* | Acetilcholinesterase | NM_015831.2 |
| *ACVR1* | Activin A receptor type I | NM_001105.4 |
| *ADA* | Adenosine deaminase | NM_000022.2 |
| *ADAMTS1* | ADAM metallopeptidase with thrombospondin type 1 motif 1 | NM_006988.3 |
| *ADCYAP1* | Adenylate cyclase activating polypeptide 1 | NM_001099733.1 |
| *AGT* | Angiotensinogen | NM_000029.3 |
| *AGTR1* | Angiotensin II receptor type 1 | NM_031850.3 |
| *AKT* | V-akt murine thymoma viral oncogene homolog 1 | NM_001014432.1 |
| *ALOX15* | Arachidonate 15-lipoxygenase | NM_001140.3 |
| *ALPP* | Placental alkaline phosphatase | NM_001632.3 |
| *AMHR2* | Anti-Mullerian hormone receptor type II | NM_020547.2 |
| *AMN* | Amnion associated transmembrane protein | NM_030943.3 |
| *ANTXR2* | Anthrax toxin receptor 2 | NM_001145794.1 |
| *ANXA5* | Annexin A5 | NM_001154.3 |
| *APOE* | Apolipoprotein E | NM_001302691.1 |
| *AQP9* | Aquaporin 9 | NM_020980.3 |
| *AR* | Androgen receptor | NM_000044.3 |
| *AREG* | Amphiregulin | NM_001657 |
| *ARNT* | Aryl hydrocarbon receptor nuclear translocator | NM_001668.3 |
| *BAX* | BCL2-associated X protein | NM_004324.3 |
| *BCL2* | B-cell CLL/lymphoma 2 | NM_000633.2 |
| *BMP2* | Bone morphogenetic protein 2 | NM_001200.2 |
| *BMP4* | Bone Morphogenetic Protein 4 | NM_130850.3 |
| *BMP7* | Bone Morphogenetic Protein 7 | NM_001719.2 |
| *BMP8A* | Bone Morphogenetic Protein 8a | NM_181809.3 |
| *BOK* | BCL2-related ovarian killer | NM_032515.4 |
| *BSG* | Basigin | NM_001728.3 |
| *BTC* | Betacellulin | NM_001729.2 |
| *BTF3* | Basic transcription factor 3 | NM_001037637.1 |
| *C3* | Complement component 3 | NM_000064.2 |
| *C4BP* | Complement component 4 binding protein | NM_000715.3 |
| *C5AR1* | Complement component 5a receptor 1 | NM_001736.3 |
| *CALCA* | Calcitonin-related polypeptide alpha | NM_001741.2 |
| *CASP10* | Caspase 10, apoptosis-related cysteine peptidase | NM_032977.3 |
| *CASP12* | Caspase 12, apoptosis-related cysteine peptidase | NM_001191016.1 |
| *CASP3* | Caspase 3, apoptosis-related cysteine peptidase | NM_004346.3 |
| *CASP6* | Caspase 6, apoptosis-related cysteine peptidase | NM_001226.3 |
| *CASP8* | Caspase 8, apoptosis-related cysteine peptidase | NM_001080125.1 |
| *CASP9* | Caspase 9, apoptosis-related cysteine peptidase | NM_001229.3 |
| *CBS* | Cystathionine-beta-synthase | NM_000071.2 |
| *CD44* | CD44 molecule | NM_000610.3 |
| *CD46* | CD46 molecule | NM_172359.2 |
| *CD55* | CD55 Molecule, Decay Accelerating Factor For Complement | NM_001114752.1 |
| *CDH1* | Cadherin 1 type 1, E-cadherin (epithelial) | NM_004360.3 |
| *CDH11* | Cadherin 11 type 2, OB-cadherin (osteoblast) | NM_001797.2 |
| *CDKN1C* | Cyclin-Dependent Kinase Inhibitor 1C | NM_000076.2 |
| *CDKN2B* | Cyclin-Dependent Kinase Inhibitor 2B | NM_004936.3 |
| *CGB5* | Chorionic gonadotropin, beta polypeptide 5 | NM_033043.1 |
| *CGB8* | Chorionic gonadotropin, beta polypeptide 8 | NM_033183.2 |
| *CLDN4* | Claudin 4 | NM_001305.3 |
| *COL6A3* | Collagen, type VI, alpha 3 | NM_004369.3 |
| *CPB2* | Carboxypeptidase B2 | NM_001872.3 |
| *CR1* | Complement Component (3b/4b) Receptor 1 | NM_000651.4 |
| *CSF1* | Colony stimulating factor 1 | NM_172212.2 |
| *CTLA4* | Cytotoxic T-Lymphocyte-Associated Protein 4 | NM_005214.4 |
| *CTNNA3* | Catenin alpha 3 | NM_001127384.1 |
| *CXCL8* | Chemokine (C-X-C Motif) Ligand 8 | NM_000584.3 |
| *CYP19A1* | Cytochrome P450 family 19 subfamily A, polypeptide 1 | NM_031226.2 |
| *CYP2D6* | Cytochrome P450 family 2 subfamily D, polypeptide 6 | NM_000106.5 |
| *EGFR* | Epidermal growth factor receptor | NM_005228.3 |
| *EOMES* | Eomesodermin | NM_005442.3 |
| *EPAS1* | Endothelial PAS domain protein 1 | NM_001430.4 |
| *ERBB4* | v-erb-b2 avian erythroblastic leukemia viral oncogene homolog 4 | NM_005235.2 |
| *EREG* | Epiregulin | NM_001432.2 |
| *ESR1* | Estrogen receptor 1 | NM_001122742.1 |
| *ESR2* | Estrogen Receptor 2 (ER Beta) | NM_001437.2 |
| *F13A1* | Coagulation factor XIII, A1 polypeptide | NM_000129.3 |
| *F2* | Prothrombin | NM_000506.3 |
| *F5* | Coagulation factor V (proaccelerin, labile factor) | NM_000130.4 |
| *FAS* | Fas cell surface death receptor | NM_000043.4 |
| *FGA* | Fibrinogen alpha chain | NM_000508.3 |
| *FGF9* | Fibroblast growth factor 9 | NM_002010.2 |
| *FGFR2* | Fibroblast growth factor receptor 2 | NM_022970.3 |
| *FGG* | Fibrinogen gamma chain | NM_021870.2 |
| *FKBP4* | FK506 binding protein 4 | NM_002014.3 |
| *FLT1* | Fms-related tyrosine kinase 1 | NM_002019.4 |
| *FOXA2* | Forkhead box A2 | NM_021784.4 |
| *FOXD1* | Forkhead box D1 | NM_004472.2 |
| *FOXP3* | Forkhead box P3 | NM_014009.3 |
| *FZD4* | Frizzled class receptor 4 | NM_012193.3 |
| *GDF15* | Growth Differentiation Factor 15 | NM_004864.2 |
| *GHR* | Growth hormone receptor | NM_001242399.2 |
| *GPC1* | Glypican 1 | NM_002081.2 |
| *GPX3* | Glutathione peroxidase 3 | NM_002084.3 |
| *GRP* | Gastrin-releasing peptide | NM_002091.3 |
| *GSTP1* | Glutathione S-transferase pi 1 | NM_000852.3 |
| *GSTT1* | Glutathione S-transferase theta 1 | NM_000853.3 |
| *HDC* | Histidine decarboxylase | NM_002112.3 |
| *HLA-C* | Major histocompatibility complex, class I, C | NM_002117.5 |
| *HLA-DQB1* | Major histocompatibility complex, class II, DQ beta 1 | NM_001243961.1 |
| *HLA-DRB1* | Major histocompatibility complex, class II, DR beta 1 | NM_002124.3 |
| *HLA-DRB5* | Major histocompatibility complex, class II, DR beta 5 | NM_002125.3 |
| *HLA-E* | Major histocompatibility complex, class I, E | NM_005516.5 |
| *HLA-G* | Major histocompatibility complex, class I, G | NM_002127.5 |
| *HMX3* | H6 family homeobox 3 | NM_001105574.1 |
| *HOXA10* | Homeobox A10 | NM_018951.3 |
| *HOXA11* | Homeobox A11 | NM_005523.5 |
| *HRH1* | Histamine Receptor H1 | NM_001098213.1 |
| *HRH2* | Histamine Receptor H2 | NM_001131055.1 |
| *ICAM1* | Intercellular adhesion molecule 1 | NM_000201.2 |
| *IDO1* | Indoleamine 2,3-dioxygenase 1 | NM_002164.5 |
| *IDO2* | Indoleamine 2,3-dioxygenase 2 | NM_194294.2 |
| *IFNA1* | Interferon, alpha 1 | NM_024013.2 |
| *IFNG* | Interferon gamma | NM_000619.2 |
| *IFNGR1* | Interferon gamma receptor 1 | NM_000416.2 |
| *IFNW1* | Interferon, omega 1 | NM_002177.1 |
| *IGF1* | Insulin-like growth factor 1 | NM_001111285.1 |
| *IGFBP1* | Insulin-like growth factor binding protein 1 | NM_000596.2 |
| *IHH* | Indian hedgehog | NM_002181.3 |
| *IL10* | Interleukin 10 | NM_000572.2 |
| *IL11* | Interleukin 11 | NM_000641.3 |
| *IL11RA* | Interleukin 11 receptro alpha | NM_001142784.2 |
| *IL12B* | Interleukin 12B | NM_002187.2 |
| *IL15* | Interleukin 15 | NM_000585.4 |
| *IL17RB* | Interleukin 17 receptor B | NM_018725.3 |
| *IL18* | Interleukin 18 | NM_001562.3 |
| *IL1A* | Interleukin 1, Alpha | NM_000575.3 |
| *IL1R2* | Interleukin 1 receptor, type II | NM_004633.3 |
| *IL1RL1* | Interleukin 1 receptor-like 1 | NM_016232.4 |
| *IL1RN* | Interleukin 1 receptor antagonist | NM_173841.2 |
| *IL2* | Interleukin 2 | NM_000586.3 |
| *IL25* | Interleukin 25 | NM_022789.3 |
| *IL2RA* | Interleukin 2 receptor, alpha | NM_000417.2 |
| *IL33* | Interleukin 33 | NM_033439.3 |
| *IL4* | Interleukin 4 | NM_000589.3 |
| *IL6* | Interleukin 6 | NM_000600.3 |
| *IL6R* | Interleukin 6 Receptor | NM_000565.3 |
| *IL6ST* | Interleukin 6 signal transducer | NM_002184.3 |
| *INHBA* | Inhibin, Beta A | NM_002192.2 |
| *IRF1* | Interferon regulatory factor 1 | NM_002198.2 |
| *ITGA5* | Integrin, alpha 5 | NM_002205.2 |
| *ITGB3* | Integrin, beta 3 | NM_000212.2 |
| *JAK2* | Janus kinase 2 | NM_004972.3 |
| *KDR* | Kinase insert domain receptor | NM_002253.2 |
| *KIR2DL1* | Killer cell immunoglobulin-like receptor, two domains, long cytoplasmic tail, 1 | NM_014218.2 |
| *KIR2DL3* | Killer cell immunoglobulin-like receptor, two domains, long cytoplasmic tail, 3 | NM_015868.2 |
| *KIR2DS1* | Killer cell immunoglobulin-like receptor, two domains, short cytoplasmic tail, 1 | NM_014512.1 |
| *KLF9* | Kruppel-like factor 9 | NM_001206.2 |
| *LEFTY2* | Left-Right Determination Factor 2 | NM_003240.3 |
| *LEP* | Leptin | NM_000230.2 |
| *LHCGR* | Luteinizing hormone/choriogonadotropin receptor | NM_000233.3 |
| *LIF* | Leukemia inhibitory factor | NM_002309.4 |
| *LIFR* | Leukemia inhibitory factor receptor alpha | NM_001127671.1 |
| *LPAR3* | Lysophosphatidic acid receptor 3 | NM_012152.2 |
| *MCAM* | Melanoma cell adhesion molecule | NM_006500.2 |
| *MCL1* | Myeloid cell leukemia 1 | NM_021960.4 |
| *MICA* | MHC class I polypeptide-related sequence A | NM_001177519.2 |
| *MIF* | Macrophage migration inhibitory factor | NM_002415.1 |
| *MMP10* | Matrix Metallopeptidase 10 | NM_002425.2 |
| *MMP12* | Matrix Metallopeptidase 12 | NM_002426.4 |
| *MMP19* | Matrix Metallopeptidase 19 | NM_002429.4 |
| *MMP2* | Matrix metallopeptidase 2 | NM_004530.4 |
| *MMP26* | Matrix Metallopeptidase 26 | NM_021801.3 |
| *MMP9* | Matrix Metallopeptidase 9 | NM_004994.2 |
| *MSX1* | Msh Homeobox 1 | NM_002448.3 |
| *MTHFR* | Methylenetetrahydrofolate reductase | NM_005957.4 |
| *MTR* | 5-methyltetrahydrofolate-homocysteine methyltransferase | NM_000254.2 |
| *MUC1* | Mucin 1 | NM_001204286.1 |
| *NCOA1* | Nuclear Receptor Coactivator 1 | NM_003743.4 |
| *NCOA2* | Nuclear receptor coactivator 2 | NM_006540.2 |
| *NDP* | Norrie Disease | NM_000266.3 |
| *NOS3* | Endothelial nitric oxide synthase 3 | NM_000603.4 |
| *NR2F2* | Nuclear receptor subfamily 2, group F, member 2 | NM_021005.3 |
| *NR3C1* | Nuclear Receptor Subfamily 3, Group C, Member 1 (Glucocorticoid Receptor) | NM_001024094.1 |
| *PAEP* | Progestagen-associated endometrial protein | NM_001018049.1 |
| *PCYT1A* | Phosphate cytidylyltransferase 1, choline, alpha | NM_005017.2 |
| *PER1* | Period circadian clock 1 | NM_002616.2 |
| *PGR* | Progesterone receptor | NM_000926.4 |
| *PLA2G4A* | Phospholipase A2, group IVA | NM_024420.2 |
| *PPARG* | Peroxisome proliferator-activated receptor gamma | NM_015869.4 |
| *PRL* | Prolactin | NM_000948.5 |
| *PRLR* | Prolactin receptor | NM_000949.5 |
| *PROCR* | Endothelial protein C receptor | NM_006404.4 |
| *PROKR1* | Endocrine-Gland-Derived Vascular Endothelial Growth Factor | NM_138964.2 |
| *PROKR2* | Prokineticin 2 | NM_144773.3 |
| *PROZ* | Protein Z, vitamin K-dependent plasma glycoprotein | NM_001256134.1 |
| *PTEN* | Phosphatase and tensin homolog | NM_000314.4 |
| *PTGFR* | Prostaglandin F Receptor | NM_000959.3 |
| *PTGIS* | Prostaglandin I2 (prostacyclin) synthase | NM_000961.3 |
| *PTGS1* | Prostaglandin-Endoperoxide Synthase 1 | NM_000962.2 |
| *PTGS2* | Prostaglandin-endoperoxide synthase 2 | NM_000963.2 |
| *PTPN11* | Protein tyrosine phosphatase, non-receptor type 11 | NM_002834.3 |
| *PTX3* | Pentraxin 3 | NM_002852.3 |
| *PZP* | Pregnancy-zone protein | NM_002864.2 |
| *RAMP1* | Receptor (G protein-coupled) activity modifying protein 1 | NM_005855.3 |
| *RGS2* | Regulator of G-protein signaling 2 | NM_002923.3 |
| *RHOB* | Ras homolog family member B | NM_004040.2 |
| *RPS6KA3* | Ribosomal protein S6 kinase | NM_004586.2 |
| *S1PR3* | Sphingosine-1-phosphate receptor 3 | NM_005226.3 |
| *SDC2* | Syndecan 2 | NM_002998.3 |
| *SELL* | Selectin L | NM_000655.4 |
| *SERPINB2* | Serpin peptidase inhibitor, clade B (ovalbumin), member 2 | NM_002575.2 |
| *SERPINC1* | Serpin peptidase inhibitor, clade C (antithrombin), member 1 | NM_000488.3 |
| *SERPINE1* | Plasminogen activator inhibitor type 1 | NM_000602.4 |
| *SLC13A1* | Solute carrier family 13 (sodium/sulfate symporter), member 1 | NM_022444.3 |
| *SLC2A1* | Solute Carrier Family 2 (Facilitated Glucose Transporter), member 1 | NM_006516.2 |
| *SLCO2A1* | Solute carrier organic anion transporter family member 2A1 | NM_005630.2 |
| *SMAD2* | SMAD Family Member 2 | NM_005901.5 |
| *SMAD4* | SMAD Family Member 4 | NM_005359.5 |
| *SNED1* | Sushi, nidogen and EGF-like domains 1 | NM_001080437.1 |
| *SPP1* | Secreted phosphoprotein 1 | NM_001251830.1 |
| *SRC* | V-src avian sarcoma (Schmidt-Ruppin A-2) viral oncogene homolog | NM_005417.3 |
| *STAT3* | Signal transducer and activator of transcription 3 | NM_139276.2 |
| *STAT5B* | Signal transducer and activator of transcription 5B | NM_012448.3 |
| *SYCP3* | Synaptonemal complex protein 3 | NM_153694.4 |
| *TEK* | TEK tyrosine kinase, endothelial | NM_000459.4 |
| *TFPI* | Tissue factor pathway inhibitor | NM_006287.4 |
| *TGFB1* | Transforming growth factor, beta 1 | NM_000660.4 |
| *TGFBR1* | Transforming Growth Factor, Beta Receptor 1 | NM_004612.2 |
| *THBD* | Thrombomodulin | NM_000361.2 |
| *TIMP1* | TIMP metallopeptidase inhibitor 1 | NM_003254.2 |
| *TIMP2* | TIMP metallopeptidase inhibitor 2 | NM_003255.4 |
| *TIMP3* | TIMP metallopeptidase inhibitor 3 | NM_000362.4 |
| *TIMP4* | TIMP metallopeptidase inhibitor 4 | NM_003256.3 |
| *TLR3* | Toll-like receptor 3 | NM_003265.2 |
| *TNC* | Tenascin C | NM_002160.3 |
| *TNF* | Tumor necrosis factor | NM_000594.3 |
| *TNFSF15* | Tumor necrosis factor (ligand) superfamily, member 15 | NM_005118.3 |
| *TNFSF8* | Tumor necrosis factor (ligand) superfamily, member 8 | NM_001244.3 |
| *TP53* | Tumor protein 53 | NM_001126114.2 |
| *TRAF3IP1* | TNF receptor-associated factor 3 interacting protein 1 | NM_015650.3 |
| *TRB* | T cell receptor beta locus | NG_001333.2 |
| *TRO* | Trophinin | NM_001039705.1 |
| *TYMP* | Thymidine phosphorylase | NM_001257989.1 |
| *TYROBP* | TYRO protein tyrosine kinase binding protein | NM_003332.3 |
| *ULBP1* | UL16 binding protein 1 | NM_025218.2 |
| *VCAM1* | Vascular cell adhesion molecule 1 | NM_001078.3 |
| *VEGFA* | Vascular Endothelial Growth Factor A | NM_001025366.2 |
| *WNT4* | Wingless-type MMTV integration site family, member 4 | NM_030761.4 |
| *WNT5A* | Wingless-type MMTV integration site family, member 5a | NM_003392.4 |
| *WNT6* | Wingless-type MMTV integration site family, member 6 | NM_006522.3 |
| *WNT7A* | Wingless-type MMTV integration site family, member 7A | NM_004625.3 |
| *ZEB1* | Zinc finger E-box binding homeobox 1 | NM_001174096.1 |
