# Supplementary material for: Novel genes and mutations in patients affected by recurrent pregnancy loss
Source: PLoS One. 2017 Oct 10;12(10):e0186149. doi: 10.1371/journal.pone.0186149 (PMC5634651; doi:10.1371/journal.pone.0186149)
Supplement: S1 Methods — (DOCX) [file pone.0186149.s004.docx]

**Novel genes and mutations in patients affected by recurrent pregnancy loss**

Paula Quintero-Ronderos, Eric Mercier, Michiko Fukuda, Ronald González, Carlos Fernando Suárez, Manuel Alfonso Patarroyo, Daniel Vaiman , Jean-Christophe Gris and Paul Laissue

**Supplementary Methods**

**Library preparation and sequencing**

Total DNA from patients was extracted from blood leucocytes by conventional salting-out procedure. Thirty-one samples (Pt-1 throw Pt-31) were used for Illumina NGS sequencing as following. Library preparation and sequencing were performed using SureSelectXT Reagent Kit, HSQ, 16 (Catalog # G9611A). 200 nanograms of genomic DNA from each sample was made up to 50 ul with Low TE and sonicated on Covaris S220 system to fragment DNA into size ranging between 150 to 400 bp. The size distribution was checked by running an aliquot of the sample on an Agilent HS Bioanalyzer Chip. Subsequently, genomic DNA libraries were constructed according to the SureSelectXT Target Enrichment System for Illumina Paired-End Sequencing Library manual (Part # G7530-90000 Version B.1, December 2014). DNA was subjected to a series of enzymatic reactions that repair frayed ends, phosphorylate the fragments, add a single nucleotide A overhang and ligate adaptors. After ligation, PCR amplification (10 cycles) was performed to enrich the adaptor-ligated fragments. The prepared library was then kept for hybridization as outlined in SureSelectXT Target Enrichment System for Illumina Paired-End Sequencing Library manual. Hybridized library fragments were isolated by magnetic capture using Magnetic Streptavidin coated beads (T1). The captured library beads were resuspended in 15 ul of NFW. PCR amplification (12 cycles) was carried out according to the protocol to amplify the captured library. Index was added at this stage. Clean up was done using HighPrep PCR clean up system. Captured Library was quantified using Qubit fluorometer. An aliquot of the captured library was run on a Agilent High Sensitivity Bioanalyzer Chip. The Illumina-compatible ready libraries were denatured and sequenced on the Illumina HiSeq 4000 using sequencing by synthesis technology for 150 bases paired end chemistry. DNA library fragments were diluted, denatured and hybridized to a lawn of oligonucleotides immobilized on the flow cell surface. Hybridized DNA template was amplified using immobilized oligonucleotides as primers. Each hybridized template through the process of isothermal bridge amplification resulted in the formation of clusters comprised of roughly 1000 clonal copies. Sequencing was performed by synthesis (SBS) technology using four fluorescent labeled nucleotides to sequence each cluster on the flow cell surface in parallel. During each sequencing cycle, a single labeled deoxynucleotide triphosphate (dNTP) was added and clusters were imaged. The fluorescent dye and blocker was cleaved off and the next complementary base was added to the nucleic acid chain and imaged. 150 of such cycles were performed which corresponds to 150 bases sequenced. Individual bases were called directly from signal intensity measurements during each cycle. These cycles comprised Read 1 of the sequencing run. Read 1 was completed, a second set of cluster generation took place on the HiSeq. Once the clusters were generated, sequencing was performed again on the reverse strand (Read 2) for an additional 150 cycles.

Thus, total of bases read is 300 bases i.e. 150 bases for Read 1 and 150 bases for Read 2.

Once sequencing was completed, the raw data was extracted from the server using the proprietary Illumina pipeline software to obtain FASTQ files. Quality check of raw data was performed using a Genotypic Propertiary scripts. The goal of read trimming is the removal of undesired base calls at the 3' end of a read, to remove the adapter sequence and the lower quality 3' ends. The low quality and the adapter trimming is done with the Genotypic proprietary script for adapter and low quality filtering. The reads post processing were aligned the HG19 reference with Bwa mem program. Further to the alignment, the variants were identified with Samtools and Bcftools. Only for those regions which are present in the Sureselect-Exome-50Mb-hg19_revA (target) region. The only variants which passed the threshold of minimum coverage of 20x (twenty reads covering that particular base) and minimum quality of Q30 had been reported. The variants detected were further annotated using the Variant studio to give location (intronic/exonic/utr), gene name, protein change, function and dbSNP Id (from the dbSNP database 137) and Variant Effect Predictor for SIFT and Polyphen prediction.

Eighteen samples (Pt-32-Pt-49) were used for Ion Proton NGS sequencing as following. Library preparation was performed using Ion TargetSeq™ Exome Enrichment for the Ion Proton™ System (Part # MAN0006730, Revision 5.0). Genomic DNA (3ug) was fragmented using Ion Shear Enzyme. The resulting fragmented DNA was cleaned up using HighPrep PCR clean up system (Magbio#AC-60050). The size distribution was checked by running an aliquot of the sample on 2% Egel.

Subsequently, genomic DNA library was constructed according to Ion TargetSeq™ Exome Enrichment for the Ion Proton™ System (Part # MAN0006730, Revision 5.0). End-repair and adapter ligation have done according to the protocol. The sample was cleaned using HighPrep PCR (Magbio#AC-60050) beads, size-selected on gel at ~280bp and eluted using MinElute column. PCR amplification (10 cycles) was performed to enrich the adaptor-ligated fragments. For capture, 500ng of prepared library was concentrated using a Vacuum concentrator (Eppendorf). The library was then hybridized to Ion TargetSeq™ Exome Probe Pool at 47°C for 66 hours. Hybridized library fragments were isolated by magnetic capture using Dynal M-270 streptavidin coated beads (Invitrogen). PCR amplification (8 cycles) was carried out to amplify the captured library and cleaned up using HighPrep PCR (Magbio#AC-60050) beads. An aliquot of the captured library was run on Agilent High Sensitivity Bioanalyzer Chip. Real time PCR validation was performed with pre and post capture libraries to observe the capture efficiency. The purified, exome-enriched library was then used to prepare clonally amplified templated Ion PI™ Ion Sphere™ Particles (ISPs) for sequencing on an Ion PI™ Chip to obtain the necessary data coverage. The samples were sequenced with Ion Proton™ Sequencer of Torrent suite v3.6. Once the sequencing is done, the raw reads undergo the process of trimming and filtering to get only the high quality reads. Only those which passes these filters came out in the FASTQ format. The raw reads obtained are aligned to the reference HG19 with the TMAP algorithm. The variants detected with the variant caller plugin were further annotated using the Ion Reporter 4.2 to give location (intronic/exonic/utr), gene name, protein change, function and dbSNP Id (from the dbSNP database 137) and Variant effect predictor for SIFT and Polyphen prediction. Library preparation and sequencing were carried out at Genotypic Technology’s Genomics facility (Bangalore, Karnataka, India).

A subset of 234 RPL (RPL-234) candidate genes (**Supplementary Table 2**) was generated by consulting PubMed, MGI-Jackson Laboratory, Genecards, and Illumina NextBio websites for filtering NGS data (Excel and R software programming) potentially related to the patients’ phenotypes. The **Supplementary Figure S1** describes the methodological pipeline for creating the 234-RPL subset.

More precisely, the 234 RPL candidate genes (RPL-234 subset) were selected by a systematic review of the literature and public internet databases including information on gene expression and function. We looked for genes with relevant functions in the implantation using the PubMed database and the MeSH words: *Recurrent spontaneous abortion* OR *RSA* OR *implantation* AND *genetics*. The following keywords were also used for interrogating this database: embryo implantation, endometrium physiology, abnormal embryo attachment, embryo implantation failure, impaired embryo implantation, abnormal spontaneous abortion rate, abnormal decidualisation, abnormal post-implantation uterine environment, endometrium inflammation, abnormal uterine receptivity, recurrent spontaneous abortion, recurrent pregnancy loss, miscarriage, and the genetics of miscarriage. We included reports describing significant statistical associations between variants carried by candidate genes and the RPL phenotype, and genes displaying functional effects related to the disease pathogenesis. In parallel, we explored the MGI Jackson Laboratory database for identifying KO/KI murine models affected by embryonic resorption (a phenotype analogous to RPL) phenotypes, such as *Abnormal embryo attachment, Failure of embryo implantation, Impaired embryo implantation, Abnormal spontaneous abortion rate, Abnormal decidualization, Abnormal postimplantation uterine environment, Endometrium inflammation* and *Abnormal uterine receptivity*. These steps led to the creation of a preliminary list of candidate genes. The list was thereafter enriched with information contained in three databases including gene expression and function: GeneCards (<http://www.genecards.org/>), Illumina NextBio (<http://www.nextbio.com/b/nextbioCorp.nb>) and PubMed (MeSH words: *Pregnancy* OR *Gestation* OR *Embryo Implantation* OR *Decidualization* AND “*candidate gene name*”). We finally selected the genes displaying relevant information on implantation in all databases.

Sequence variants (synonymous and non-synonymous) in the RPL-234 subset reported as novel (lacking from the 1000 genome sequence database and the Exome Variant Server-EVS) were selected for subsequent analysis. Variants having a potential effect at sequence protein level (e.g. missense, nonsense, splice site, frameshift) were then filtered for downstream analysis. Sequences of mutant proteins, issued from the RPL-234 analysis, presenting amino acid variations (point mutations) were manually aligned (ClustalW software) with those from orthologous species. Sequence variants involving residues strictly conserved (C+) during evolution were checked by PCR/Sanger sequencing. One splice-site sequence variant was also checked by direct sequencing. Technical conditions for PCR/sequencing assays, including oligonucleotide sequences, are available upon request.

We used SIFT, PolyPhen2, Mutation Taster and Mutpred software to predict at protein level potential deleterious effects caused by C+ mutations. We have chosen these programs as they have demonstrated high levels of positive prediction values ^1,2^.

*Structure preparation/modelling and Fragment Molecular Orbital (FMO) calculations*

MMP-10 (pdb code 3V96) and FGA (pdb code 1FZD) crystal structures (WT) and their respective mutants (MT) were studied ^3,4^. The UCSF Chimera *swapaa* function was used to make amino acid substitutions in crystal structures, using the Dunbrack backbone-dependent rotamer library ^5,6^. The Poisson-Boltzmann method was used for calculating the residues’ protonation states, using the H++ web server and the same pH described for crystallisation (MMP-10, pH 6.5; FGA, pH 5.5) ^7^. Hydrogen atoms were optimised using the PM7 method and the conductor-like screening model (COSMO) as an implicit solvent model with fixed heavy atoms in their crystallographic positions (MOPAC2016 software) ^8^.

The FMO method was used for studying the effects of amino acid substitutions ^9^. This approach allows a comprehensive evaluation of the kinds and the amount of variations caused by mutations. This *ab initio* quantum method enables an accurate evaluation of large molecular systems by means of a partition scheme (fragments). Total interaction energy can be decomposed into electrostatic, repulsion, charge transfer, dispersion and solvation terms by using pair interaction decomposition analysis (PIEDA) for each fragment pair ^10^. The FMO method (version 5.2) implementing GAMESS 2016 software and the Hartree Fock (HF) theory with the 6-31G* basis set was used ^11^. Solvent effects were included with the polarizable continuum model (PCM). Only residues within a ≤ 10Å radius around the mutant residue were included in the FMO calculations for reducing computational costs. Grimme’s dispersion model D3 was used for correcting all HF energies ^12^. A fragmentation scheme was created considering ions for MMP-10 ^13^. FGA models were fragmented using Facio v. 19.2.1 ^14^. Interactions between fragments having a ≥3kcal/mol absolute value were considered significant ^15^.

**References**

1. Thusberg J, Olatubosun A, Vihinen M. Performance of mutation pathogenicity prediction methods on missense variants. *Hum Mutat*. 2011;32(4):358-368. doi:10.1002/humu.21445.

2. Walters-Sen LC, Hashimoto S, Thrush DL, et al. Variability in pathogenicity prediction programs: impact on clinical diagnostics. *Mol Genet Genomic Med*. 2015;3(2):99-110. doi:10.1002/mgg3.116.

3. Batra J, Robinson J, Soares AS, Fields AP, Radisky DC, Radisky ES. Matrix Metalloproteinase-10 (MMP-10) Interaction with Tissue Inhibitors of Metalloproteinases TIMP-1 and TIMP-2: binding studies and crystal structure. *J Biol Chem*. 2012;287(19):15935-15946. doi:10.1074/jbc.M112.341156.

4. Spraggon G, Applegate D, Everse SJ, et al. Crystal structure of a recombinant alphaEC domain from human fibrinogen-420. *Proc Natl Acad Sci U S A*. 1998;95(16):9099-9104.

5. Dunbrack RL. Rotamer libraries in the 21st century. *Curr Opin Struct Biol*. 2002;12(4):431-440.

6. Pettersen EF, Goddard TD, Huang CC, et al. UCSF Chimera?A visualization system for exploratory research and analysis. *J Comput Chem*. 2004;25(13):1605-1612. doi:10.1002/jcc.20084.

7. Gordon JC, Myers JB, Folta T, Shoja V, Heath LS, Onufriev A. H++: a server for estimating pKas and adding missing hydrogens to macromolecules. *Nucleic Acids Res*. 2005;33(Web Server):W368-W371. doi:10.1093/nar/gki464.

8. Stewart JJ. MOPAC: a semiempirical molecular orbital program. *J Comput Aided Mol Des*. 1990;4(1):1-105.

9. Fedorov DG, Nagata T, Kitaura K. Exploring chemistry with the fragment molecular orbital method. *Phys Chem Chem Phys*. 2012;14(21):7562. doi:10.1039/c2cp23784a.

10. Fedorov DG, Kitaura K. Pair interaction energy decomposition analysis. *J Comput Chem*. 2007;28(1):222-237. doi:10.1002/jcc.20496.

11. Schmidt MW, Baldridge KK, Boatz JA, et al. General atomic and molecular electronic structure system. *J Comput Chem*. 1993;14(11):1347-1363. doi:10.1002/jcc.540141112.

12. Grimme S, Ehrlich S, Goerigk L. Effect of the damping function in dispersion corrected density functional theory. *J Comput Chem*. 2011;32(7):1456-1465. doi:10.1002/jcc.21759.

13. Hitaoka S, Chuman H, Yoshizawa K. A QSAR study on the inhibition mechanism of matrix metalloproteinase-12 by arylsulfone analogs based on molecular orbital calculations. *Org Biomol Chem*. 2015;13(3):793-806. doi:10.1039/C4OB01843E.

14. Suenaga M. Facio: New Computational Chemistry Environment for PC GAMESS. *J Comput Chem Japan*. 2005;4(1):25-32. doi:10.2477/jccj.4.25.

15. Heifetz A, Chudyk EI, Gleave L, et al. The Fragment Molecular Orbital Method Reveals New Insight into the Chemical Nature of GPCR–Ligand Interactions. *J Chem Inf Model*. 2016;56(1):159-172. doi:10.1021/acs.jcim.5b00644.
