# Supplementary material for: Novel genes and mutations in patients affected by recurrent pregnancy loss
Source: PLoS One. 2017 Oct 10;12(10):e0186149. doi: 10.1371/journal.pone.0186149 (PMC5634651; doi:10.1371/journal.pone.0186149)
Supplement: S1 Results — (DOCX) [file pone.0186149.s005.docx]

**Novel genes and mutations in patients affected by recurrent pregnancy loss**

Paula Quintero-Ronderos, Eric Mercier, Michiko Fukuda, Ronald González, Carlos Fernando Suárez, Manuel Alfonso Patarroyo, Daniel Vaiman , Jean-Christophe Gris and Paul Laissue

**Supplementary Results**

The FMO method was used for studying FGA (fibrinogen alpha chain) (**Figure 2**) and MMP-10 (matrix metallopeptidase 10) (**Figure 3**) WT and MT structures regarding the effect of amino acid substitutions. We have selected these proteins for structural analysis because regions carrying putative deleterious mutations were previously crystallized.

**Figure 2A and 3A** show the calculated values for FGA and MMP-10 models, respectively. The phenylalanine mutated residue located at position 685 corresponds, into the crystallographic structure, to the amino acid located at position 666. Therefore, for FMO approach we will use Phe666 instead Phe685.

Regarding FGA, FMO analysis showed that Phe666 being replaced by Cys666 suggested a deleterious effect on stabilising interactions. Two major interactions were found (**Figure 2**) concerning the WT protein. One hydrogen bond was formed between Phe666 backbone and Tyr673 side chain. An additional non-classical H-bond CH-π interaction was formed between Phe666 and Arg659. Two more significant interactions were detected; Phe666 formed a non-classical H-bond with Trp689 and Phe677. All these interactions were dominated by the dispersion term. Regarding the Cys666 MT, four interactions were identified by FMO. The backbone of Cys666 formed a hydrogen bond with Tyr673 side chain and an additional hydrogen bond was formed between Cys666 side chain and Asn799 backbone. However, two destabilising interactions dominated by repulsion effects were detected by FMO: the Cys666 thiol group with Tyr673 and Asn799 side chains (**Figure 2**).

Analysis of MMP-10 model led to identifying three major interactions by means of the FMO method for residue Asp199 in WT (**Figure 3A and 3B**). One salt bridge was formed between deprotonated Asp199 and protonated Lys165. This interaction consisted of a combination of two non-covalent interactions: hydrogen bonding and electrostatic interactions. The MMP-10 protein contains a structural zinc ion (Zn^2+^) coordinated by four residues (His167, His182, His195 and Asp169) and its interaction with Asp199 is mainly driven by electrostatic interactions. This region was named Zn block. Another important interaction was found between Asp199 and protonated His178 which is dominated by the electrostatic term (**Figure 3B**). Distinct significant interactions were also detected by the FMO method. One was formed between deprotonated Asp199 and protonated Lys201 and was driven by the electrostatic term. Asp199 side chain also formed a hydrogen bond with Gly175 backbone. Additional stabilising interactions were found between Asp199 and another five residues: Asp123, Trp202, Phe173, Gly177 and Ser179 (**Figure 3E**).

FMO only identified one interaction for Asn199 MT (**Figure 3A and 3C**). It is worth noting that the salt bridge between deprotonated Asp199 and protonated Lys165 was missing due to charged Asp199 being replaced by non-charged Asn199. The interaction between Asn199 and protonated Lys165 was dominated by the electrostatic term. The remaining interactions stabilising the interaction in the WT became lost in the Asn199 MT structure.
